# The Unseen Déjà-Vu: From Erkki Huhtamo’s *Topoi* to Ken Jacobs’ Remakes: Commentary to Edwin Carels “Revisiting *Tom Tom*: Performative anamnesis and autonomous vision in Ken Jacobs’ appropriations of *Tom Tom the Piper’s Son*”

**DOI:** 10.1007/s10699-016-9516-5

**Published:** 2016-12-24

**Authors:** Wanda Strauven

**Affiliations:** Institut für Theater-, Film- und Medienwissenschaft, Goethe-Universität Frankfurt, Norbert-Wollheim-Platz 1, 60329 Frankfurt am Main, Germany

**Keywords:** Media archaeology, Topos, Deep time, Déjà-vu, Viewing position

## Abstract

This commentary on Edwin Carels’ essay “Revisiting *Tom Tom*: Performative anamnesis and autonomous vision in Ken Jacobs’ appropriations of *Tom Tom the Piper’s Son*” broadens up the media-archaeological framework in which Carels places his text. Notions such as Huhtamo’s *topos* and Zielinski’s “deep time” are brought into the discussion in order to point out the difficulty to see what there is to see and to question the position of the viewer in front of experimental films like *Tom Tom the Piper’s Son* and its remakes.

As recounted by Carels throughout his article, *Tom Tom the Piper’s Son* went through various technological “updates” over the past centuries. The original nursery rhyme about a boy who stole a pig was published in two different versions in London around 1795, but it probably existed long before that date as a popular folk tune. In 1905 the American Mutoscope & Biograph Company adapted the rhyme for the screen, placing the opening scene (or tableau) in a setting inspired by William Hogarth’s famous engraving *Southwark Fair* (1733). It is this (early) filmic *Tom Tom the Piper’s Son*, which had a duration of ca. 10 min, that is rediscovered and revisited by experimental filmmaker Ken Jacobs, first in the late 1960s and early 1970s (*Tom Tom the Piper’s Son*, 1969–1971) and then again at the beginning of the twenty-first century by means of two remakes: *Return to the Scene of the Crime* (2008) and *Anaglyph Tom (Tom with Puffy Cheeks)* (2008). As Carels reminds us, the 1733 Hogarth engraving was itself a remake, “literally mirroring the composition of the original painting of the same year”—which should be seen as a conscious act to commercialize art via technological reproduction (Carels 2016). Another important detail that Carels does not point out as such is the fact that the 1905 Biograph film stages the act of theft at a street fair, by which it re-activates the commonplace (or *topos*) of crowded outdoor/public places as being unsafe, as gatherings for thieves (even if they are innocent young boys like Tom “who [don’t] know it [is] wrong to steal”^1^).

The notion of *topos* is central to Erkki Huhtamo’s way of studying media history. Borrowed from literary scholar Ernst Robert Curtius, the concept refers to a (literary) convention or commonplace that returns periodically over time. The media-archaeological approach proposed by Huhtamo is a search for the typical or the clichéd, for those “phenomena that (re)appear and disappear and reappear over and over again and somehow transcend specific historical context” (Huhtamo 1996: p. 300). Although they often seem to emerge “unconsciously”, *topoi* are, in Huhtamo’s view, ideological constructs that can be consciously (re-)activated by the (media) industry. Like other media-archaeological approaches, the concept of *topos* is an attempt to counter the newness of new media.^2^ A *topos* is—very literally—a place where century-old ideas manifest themselves, even if, at first sight, they might not be recognized as such because they are offered to us in a new (technologically updated) package. To understand the internal dynamics of the history of old and new media, Huhtamo proposes to study the life of *topoi*. This inevitably leads to a cyclical view of history characterized not only by returns but also by (anti-Foucauldian) continuities. Such a cyclical view has been criticized by other prominent media archaeologists, such as Siegfried Zielinski and Thomas Elsaesser, who have, each in their own way, addressed media history in terms of discontinuity, singularity and fragmentation instead (Zielinksi 2006; Elsaesser 2004).

However, with respect to Carels’ analysis, Huhtamo’s notion of *topos* seems particularly relevant to me. First of all, it is important to keep in mind that Huhtamo, for his own media-archaeological project, is mostly intrigued by what he calls “peep media”. In the Hogarth engraving, the peepshow appears as well. It stands out as one of the main attractions of the fair, well identifiable, placed upfront and a bit distant from the crowded scene. Carels points out that Jacobs not only makes the connection between this early-eighteenth-century depiction of “forced perspectives” and the movies, but also refers, in the end titles of *Return to the Scene of the Crime*, to the historical importance of the Southwark Fair for the emergence of British cinema. The very first public cinema screening supposedly took place at the actual site of the fair. And, as I pointed out above, this was clearly not the safest (or most orderly) place of the borough.

The mix of optimism and anxiety that surrounds all forms of modernity throughout history is another central aspect of Huhtamo’s media-archaeological study of *topoi*, by which he wants to explain what Tom Gunning in the early 1990s described as “an uncanny sense of *déjà*-*vu*” (Gunning 1991: 185). The context of Gunning’s (false) impression of experiencing a past experience was the approaching end of the millennium with all its uncertainties and potentialities about the future. It was precisely this combination of worry and expectation that placed Gunning, so to say, in the shoes of Sigmund Freud, who at the end of the nineteenth century went through a similar kind of experience. Gunning (falsely) re-experiences (or recollects) how the then new technologies, such as the railway and the ocean liner, created huge distances between relatives or friends, distances that could be bridged again thanks to other new technologies, such as the telegraph or the telephone. In other words, Gunning’s “uncanny sense of *déjà*-*vu*” shares with Huhtamo’s *topos* the transcending of historical periods. Moreover, Huhtamo’s *topos*—as rereading of Gunning’s *déjà*-*vu*—could be connected not only to Aby Warburg’s notion of *Nachleben*, which Carels, towards his conclusion, discusses as a “temporal unconscious”, but also to the notion of “anamnesis” (i.e. the incarnation of past knowledge) that appears in Carels’ subtitle without being elucidated any further in the article.

Whereas déjà-vu is commonly understood as a (psychological) sensation or feeling rather than a mere act of viewing (or a result of such an act), its literal meaning (“already seen”) can thus be related, in a productive way, to the notion of *topos* and even, as I will try to illustrate, to Jacobs’ *Tom Tom* remakes. Let me make clear immediately that I am not so much interested here in the “already seen” of the déjà-vu as the repetition or reappearance of something recognizable or familiar, but rather in the exact opposite: that what went unnoticed during the first act of viewing, or, put differently, the *unseen* déjà-vu. The (uncanny) awareness of the return of a certain *topos* might function, like in the case of Gunning, as a direct invitation to revisit that past moment, to look for elements that were not seen (or understood) before. In the best case this should not lead to the understanding of the past through the eyes of the present, but instead entail a true digging into the various layers of that past moment without any precondition, in search of the not-yet-seen of the “already seen”. This idea of the unseen déjà-vu comes close to Zielinski’s method of excavating into the “deep time” of media, whose development—like the formation of geological strata—is unpredictable. According to Zielinksi, media history, like geology, does not follow a linear progression. Therefore, the historian does not know what he or she will hit upon when mining into its non-linear, non-progressive layers. He or she might find something new (unseen) in the old (déjà-vu) (Zielinski 2006).

Interestingly, Carels’ conclusion seems (implicitly) related to Zielinski’s notion of “deep time” when he writes that “using more contemporary media, the archeologist Jacobs mines the original images deeper, both in a structural and an iconographical sense” (Carels 2016). In his two recent remakes of *Tom Tom the Piper’s Son*, Jacobs has recourse to, respectively, electronic video-effects and 3D technology. State-of-the-art technology is not a fetishistic end in itself for the filmmaker, but a tool to excavate further, to see what he had not seen before, when he read the 1905 film for the first time(s). As Carels rightly points out, when Jacobs started his *Tom Tom* project in the late 1960s, he anticipated media archaeology not only as a (academic) discipline but also as a (artistic) practice. He dedicated himself to early cinema with a renewed, “astonished”^3^ look, as many New Film History scholars would do a decade later, during and after the famous 34th FIAF conference that took place in 1978 in Brighton, UK.^4^ What is more, Jacobs’ work was a direct inspiration for this new generation of film historians, especially for Tom Gunning. Yet Jacobs was not an isolated figure. Other experimental filmmakers should be mentioned here in order to put into perspective Jacobs’ role as a pioneering media-archaeologist. For instance, Stan Brakhage, Noël Burch and documentary film editor Dai Vaughan were equally important for the (re)discovery of early cinema. In his recount of how the “cinema of attractions” came into the world, Gunning also adds the names of Ernie Gehr and Hollis Frampton, who, around the same time, formed the so-called Chambers Street Group together with Jacobs. About this group, Gunning writes:

Each of these filmmakers not only looked carefully at films from the period of early cinema, but incorporated them into their own works, often mining the Library of Congress’ Paper Film Collection, as in Jacobs’s *Tom, Tom, the Piper’s Son* (1969) and Frampton’s *Public Domain* (1972). Speaking personally, the influence of the fresh perspective on early cinema opened up by these filmmakers played a key role in not only refocusing my attention on this period, but re-contextualizing the films, liberating them from the teleological approach that classed them as “primitive” attempts at later forms. (Gunning 2006: p. 34)

What makes Jacobs’ *Tom Tom* project unique, however, is the fact that he revisits, obsessively and exhaustively, not only the early film footage but also his own media-archaeological work by means of the two recent remakes (or “reprises”, as Carels prefers to call them). He is excavating his own excavation, by “min[ing] the original images deeper” (Carels 2016). He repeats his act of viewing to see what he (and the viewer) had not seen before.

Already in his first *Tom Tom* translation, Jacobs is concerned with making the viewer aware about the unseen déjà-vu. By showing the 1905 film again in its entirety at the end of its two-hour long examination and manipulation, Jacobs wants us to reflect on how much, as Carels puts it, “[our] gaze has altered through the radical concentration on deconstruction” (Carels 2016). We see the “already seen” again, but now totally different. The original reason for Jacobs to revisit Biograph’s *Tom Tom* can be found in the complexity of this 1905 film, especially of its opening scene, which Carels describes as “an astounding choreography of mini-dramas and micro-events, impossible to grasp in a single viewing” (Carels 2016). In his first exploration/excavation of *Tom Tom* of the late 1960s and early 1970s, Jacobs guides the viewer through an analytical and repeated viewing process to make him or her see what there is to see. In the 2008 reprises, Jacobs takes this visual exercise to an even further extreme, by focusing on all possible details and by altering the original by means of intertitles, inserts, etcetera. At the beginning of his essay, Carels wonders what Jacobs possibly would have “want[ed] the viewer to experience” (Carels 2016). While this question is somehow answered in the first part of the essay concerning the first *Tom Tom* translation, both *Return to the Scene of the Crime* and *Anaglyph Tom* seem to be made first and foremost to satisfy a very personal need of the author. Indeed, toward the end of his essay, Carels writes: “for Jacobs it is not the original status of his source material that matters, but a sense of personal discovery through visual experience” (Carels 2016). Does such an idiosyncratic project leave room for the viewer? Does the viewer see what he or she is supposed to see?

My main critique of Carels’ analysis is that his text concentrates mainly on the perspective of the maker and looses sight of the perspective of the viewer. Yet, the fundamental question seems to me exactly this: whom is Jacobs addressing with his *Tom Tom* remakes? Who is that viewer not only capable but also willing to see what there is to see, to see the unseen of the “already seen”? Both the terms “anamnesis” and “autonomous vision” that appear in the subtitle of Carels’ article might have helped to better understand Jacobs’ concerns with the (concrete) viewer. Unfortunately, Carels does not clarify these concepts nor does he relate them explicitly to Jacobs’ *Tom Tom* project (or to its reception).

Let me end with two examples of “failed” spectatorship to illustrate how problematic the assumption of an unproblematic viewing position is in this specific case. My first example pertains to the use of 3D-technology for the second remake of 2008: *Anaglyph Tom (Tom with the Puffy Cheeks)*. Carels spends some time on discussing the technique of stereoscopy, but omits to comment on *Anaglyph Tom*’s actual 3D-effect (that is, from the side of the viewer). In his review for *The New York Times*, Nathan Lee points out that this 3D-effect is rather ineffective, or almost imperceptible. Familiar with Jacobs’ previous work, Lee was prepared for an “explosion of [his] eyeballs” but this failed to occur during the viewing of *Anaglyph Tom*. He adds: “[it] proves oddly less three-dimensional than some of Mr. Jacobs’s ostensibly 2-D movies; you almost suspect him of presenting it as 3-D so that he could color his images with that jittery red-blue tint you get with old-school glasses, as opposed to creating any particular spatial effect. During the second hour I removed the glasses for periods without noting any appreciable difference in the experience.” (Lee 2009) I can only repeat the question asked by Carels that was quoted above: “what does [Jacobs] want the viewer to experience” with this 3D-version of *Tom Tom*? Does he want us to remove our anaglyph glasses in order to see something different (such as the material work on the original footage)? Is the 3D-effect just a deviation?

My second example concerns Jacobs’ original masterwork from the late 1960s and its (out-of-context) reception on the Internet at the beginning of the twenty-first century. The posted comments about a 3-minute excerpt from *Tom Tom the Piper’s Son* (1969) on YouTube are telling. Especially students complain about the torturing (or, rather, excretory) viewing process. User Milmino, for instance, writes: “I’m watching this in my lecture and its shit! You only have to put up with it for 3 min and i have to watch it for a sodding two hours….! [sic]”; likewise, user j mor comments: “i had to sit through 12 min of this shite today [sic]”. But even those who find Jacobs’ work “captivating and inspiring”, as does user kolibet, admit it is “not many people’s cup of tea”. And good will is clearly not enough for user europadd who exasperatedly asks: “Is there an explanation behind this film? What’s going on here?”^5^ The didactic dimension of Jacobs’ project, underlined by Carels in his essay, does not seem to reach its target group. Of course, YouTube is not the “right” place to watch a work like *Tom Tom the Piper’s Son*, but this is part of its technological “update” or afterlife. It is a reprise (of the déjà-vu) beyond the reach of the author. What does it mean to watch Jacobs’ masterpiece in such a reduced (and relocated^6^) form? Or does such a question not matter?

## Footnotes

1 According to the subtitle of *Anaglyph Tom (Tom with Puffy Cheeks)* (2008).

2 For a general introduction to media archaeology and its various approaches, see Strauven (2013).

3 Elsaesser (2004) refers to the principle of the New Film History as a “hermeneutics of astonishment”, paraphrasing Gunning’s expression of the “aesthetics of astonishment”.

4 On the importance of the 34^th^ FIAF conference, see Strauven (2013).

5 All comments are taken from: http://www.youtube.com/watch?v=vC-zkksO-zc (accessed on May 27, 2013). Coincidentally, the excerpt was uploaded in 2008, which is the year Jacobs revisits *Tom Tom* with new technological tools.

6 I am referring here to Francesco Casetti’s notion of the “relocation of cinema” (Casetti 2012).
